# Universal Free Meals Associated with Lower Meal Costs While Maintaining Nutritional Quality

**DOI:** 10.3390/nu13020670

**Published:** 2021-02-19

**Authors:** Michael W. Long, Keith Marple, Tatiana Andreyeva

**Affiliations:** 1 Department of Prevention and Community Health, Milken Institute School of Public Health, George Washington University, 950 New Hampshire Ave. NW, Washington, DC 20052, USA; 2 The Heller School for Social Policy and Management, Brandeis University, 415 South Street, Waltham, MA 02453, USA; keith.marple@brandeis.edu; 3 Rudd Center for Food Policy and Obesity, Department of Agricultural and Resource Economics, University of Connecticut, 1 Constitution Plaza, Hartford, CT 06103, USA; tatiana.andreyeva@uconn.edu

**Keywords:** child nutrition, meal costs, food services/legislation and jurisprudence, schools

## Abstract

The Community Eligibility Provision (CEP) of the Healthy, Hunger-Free Kids Act of 2010 allows the provision of universal free meals (UFMs) in high-poverty school areas. Participation in UFM programs, including through CEP, could reduce meal costs due to economies of scale and a lower administrative burden. We analyzed the School Nutrition and Meal Cost Study (SNMCS) data from 508 UFM-eligible schools (103 UFMs) to evaluate whether meal costs varied by UFM status. We used school-level data to address the non-random selection to UFMs with inverse probability of treatment weighting (IPTW). We estimated a generalized linear model with a log link and gamma distribution to predict meal costs by UFM status and school size. Full costs among medium and large schools were marginally lower in UFM schools for lunch (−$0.673; 95% CI: −1.395, 0.0499; *p* = 0.068) and significantly lower for breakfast (−$0.575; 95% CI: −1.077, −0.074; *p* = 0.025). UFM was not associated with meal costs among smaller schools. Healthy Eating Index scores did not vary significantly by UFMs, suggesting that lower costs could be achieved without an adverse effect on nutritional quality. This analysis is limited by the lack of identified student percentage (ISP) data needed to definitively identify CEP eligibility, although results were robust to sensitivity analyses addressing the lack of ISP data. The potential policy impact of these findings emphasizes the need for future studies that assess ISP and cost with more recent data and longitudinal designs.

## 1. Introduction

The Healthy, Hunger-Free Kids Act of 2010 (HHFKA) has had a transformational effect on the nutritional landscape of schools in the United States. The first national regulation of competitive foods, which are sold as an alternative to meals served through the School Breakfast Program (SBP) and the National School Lunch Program (NSLP), eliminated the sale of most sugary drinks and reduced the calorie content of snack foods [1]. Overhauls of the nutritional requirements for meals qualifying for reimbursement through the SBP and NSLP have led to a healthier meal program with strong programmatic adherence, improvements in student diet quality, and no increases in food waste or reductions in participation [2,3,4,5]. A recent interrupted time series analysis of the National Survey of Children’s Health data from 2003–2018 found that implementation of the HHFKA was associated with a 47% reduction in the risk of obesity among children living in households with an income at or below the federal poverty level, or 500,000 fewer children with obesity in 2018 [6].

While the improvements in the nutritional quality of school meals and rules governing the nutritional quality of competitive foods have gained much of the research and policy attention, changes in the mechanisms of school meal financing have the potential to substantially increase the access and sustainability of the programs. The Community Eligibility Provision (CEP) of the HHFKA allows local educational agencies (LEAs) in low-income areas or a subset of schools in the LEA to provide free breakfast and lunch to all students. Schools and LEAs are eligible to participate in CEP if ≥40% of students in the prior year could be certified without the use of household applications. Through the elimination of the household application process and streamlining the meal counts and claiming procedures, local school districts could substantially reduce the administrative burden and related costs [1]. In the second year of national CEP availability, more than 18,000 high-poverty schools, or half of eligible schools, chose to participate in the CEP [7]. As of 2019, almost 65% of eligible schools across the nation had implemented UFM via the CEP [8], which significantly expanded access to free nutritious school meals.

Previous studies evaluating the effect of CEP participation on student-level participation, including the pilot program evaluation, have found that the CEP is associated with 5–8% higher meal participation rates, with the largest effect among students near the eligibility cutoff [9,10,11]. Additional benefits of CEP participation include reduced absenteeism and suspension rates [12,13], and improved academic achievement and perceptions of school climate [14,15,16,17]. Research on the obesity implications of CEP participation has produced mixed results, varying from no change to reduced obesity among certain student groups [14,18,19]. While the evidence of beneficial effects for children is growing, there is also a concern that the CEP may have unfavorable consequences for the financial situation of participating school districts. Federal reimbursements may fall short of covering all costs of serving universally free meals and losing revenue from meal fees. There has been just one New York–based study to date to evaluate the effect of the CEP on school meal revenues and spending, finding reduced per meal costs and a revenue gain from increased federal reimbursements that overcompensate for the reductions in food service revenues, although results were less favorable in rural areas [19].

We analyzed the School Nutrition and Meal Cost Study (SNMCS) data to evaluate whether direct meal costs and administrative costs differ across UFM and non-UFM schools. We hypothesized that UFM (and CEP) participation would be associated with lower per-meal costs due to both economies of scale from higher student participation (hence, increased federal reimbursements) and reductions in the administrative burden associated with traditional certification, collection, and processing meal payments. We hypothesized that the economies of scale due to participation would be more likely to accrue to larger schools with more scale to achieve.

The SNMCS is the most rigorous meal cost evaluation and the only national dataset that can be used to evaluate whether UFMs are associated with lower costs to school districts. Particularly in the context of COVID-19–related barriers to food access and resulting calls for new mechanisms to reduce children’s food insecurity, using the SNMCS to inform policy decisions on UFMs is critically important and time-sensitive. However, there are limitations to the SNMCS design and data availability that need to be balanced against any findings from the current analysis. This cross-sectional analysis requires a comparison group of schools against which the cost of meals in UFM (and CEP) schools can be compared. One of the most important limitations is that eligibility for the CEP cannot be determined definitively without data on the identified student percentage (ISP) at the school and district level, which are not available in the SNMCS dataset. The other threat to comparability is that schools may select into UFMs or the CEP based on measured or unmeasured characteristics that also impact the cost of producing meals. We used sample restriction, a propensity score model for UFMs, inverse probability of treatment weighting (IPTW), multivariable regression adjustment, and sensitivity analyses to address the limitations in our data.

## 2. Materials and Methods

### 2.1. Data and Design

We used data from the SNMCS, which was conducted for the U.S. Department of Agriculture, Food, and Nutrition Service (FNS) during the 2014–2015 school year [20]. The study sampled school food authorities (SFAs) (*n* = 518) operating at the school district level to be nationally representative of public SFAs offering the NSLP. In a subsample, the SNMCS included 310 SFAs and 972 schools in the cost evaluation sampling frame, which comprise the schools and SFAs analyzed in the current study. The methodology report for the SNMCS describes in detail the design, as well as sampling, recruitment, data collection, and data processing procedures [20]. Additional details about collection and analysis of the data for meal costs are available in Volume 3 of the SNMCS final report [21]. Data on the Healthy Eating Index (HEI) 2010 and score calculation are described in Volume 2 of the SNMCS final report [22].

### 2.2. Measures

The primary outcome measure for this study was the full meal cost for the SBP and NSLP. The full meal cost was estimated from a set of survey and interview instruments in the SNMCS [20]. We used reported costs as a secondary outcome in sensitivity analyses. We expected that the full costs (which included reported and unreported costs) would better capture the effect of reduced administrative burden (which may be unbudgeted and fall outside of the scope of the SFA) from the CEP and other UFM participation. We further expected that a larger proportion of the differences in reported costs would come from economies of scale, so that we would not expect substantial savings from UFMs among smaller schools unable to achieve these economies of scale. We did not have further data on the breakdown of costs (e.g., food costs vs. labor costs at the school and district levels). First, the SNMCS implemented a web-based survey to collect detailed information on the foods offered and served in school meals and afterschool snacks during a one-week target period. This web survey collected data on daily meal counts, food and beverages (whether commercially prepared or from scratch), sold in reimbursable meals, sold a la carte or to adults, left over, or wasted and other details, and, among schools in the cost evaluation subsample analyzed in the current study, additional details needed to estimate food costs per meal. Second, the school-level survey of food costs during the target week was supplemented with the SFA director and business manager on-site cost interview, which asked a range of questions needed to estimate the food, labor, other direct costs, indirect costs, off-budget costs, and costs per meal. Third, the principal cost interview, which sought to assess non-foodservice staff and non-budgeted time costs supporting the meal program, was conducted during a site visit by SNMCS staff. These costs included the following: (1) supporting applications or direct certification; (2) collecting meal payments; (3) counting and claiming reimbursable meals; (4) menu planning or nutrition education; (5) cafeteria supervision, cleaning, and management of food service staff; and (6) ordering, storing, or transporting food. These interviews were supplemented with an analysis of the SFA final expense report and follow-up interview with the SFA director and business manager.

Cost per meal was calculated by the SNMCS study using the following methods. They estimated the reported cost per meal charged to the foodservice account and unreported costs not charged to the foodservice account, such as district facilities costs not passed on to the SFA. These were summed to generate the full costs. Meal costs at the school level were estimated using the target week menu for each meal type (NLSP, SBP, CACFP, and NLSP afterschool snack). These school-level costs were adjusted to match the total annual SFA food costs. Time costs for meal preparation at the school level were estimated from the school nutrition manager cost interview and the principle cost interview. Production labor costs were allocated to reimbursable and non-reimbursable meals based on the percentages of food costs for these meals. Nonproduction school-level labor costs were allocated to meals based on the proportion of production labor costs for each meal category. SFA-level direct, indirect, reported, and unreported costs were allocated by the survey administrators to meals within schools based on the proportion of total meal costs at the school level. Full costs per meal include reported and unreported costs (food, labor, and other costs) incurred separately at the SFA and school level, with SFA-level costs applied to school-level, per-meal costs based on the proportion of total school-level meal costs within the SFA.

We created an indicator variable to show if a school operated the NSLP and SBP program under the CEP, Provision 2, or Provision 3, indicating three types of the existing UFM programs. We have expanded our focus from the CEP to UFMs to increase the sample size (CEP only participation was assessed in sensitivity analyses).

The average Healthy Eating Index (HEI) 2010 scores for NSLP lunches and SBP breakfasts were used to measure the healthfulness of school meals in each school. HEI 2010 scores ranged from 0 to 100, with higher scores indicating healthier meals. The SNMCS study team estimated the average HEI 2010 total and component scores for NSLP lunches and SBP breakfasts based on data in the Menu Survey, which were completed by school nutrition managers over one target week in the spring of SY 2014–2015. Total and component scores were generated for each school based on the average weekly menus prepared [22]. We have used the total HEI score for school lunches and the total HEI score for school breakfasts prepared during the target week.

### 2.3. Study Sample

We excluded schools in which the proportion of students eligible for free or reduced price (FRP) meal eligibility was <40%. This cutoff point was used as a proxy for school-level eligibility for the CEP, noting that some schools may participate through SFA-level eligibility and that some schools included as eligible using this cutoff point would not actually be categorized as eligible using the ISP variable unavailable here. We excluded schools that did not have complete data on any of the variables included in our propensity score models.

### 2.4. Data Analysis

To address selection among schools eligible to participate in the CEP/UFM program, we estimated propensity scores for UFM participation, and used IPTW based on these scores to adjust the sample weights [23]. IPTW is a strong method for dealing with the lack of ISP data and a clear cutoff point for CEP eligibility due to the use of weighting instead of matching and the incorporation of a broad range of predictors in the propensity score model. This means that, even if we included a non-CEP eligible school in our comparison sample based on our free and reduced price meal eligibility (FRP) category cutoff point, it would be down-weighted in the model to a degree that the non-eligible school is different from the CEP schools. We identified a broad set of school- and district-level characteristics included in the SNMCS as candidate variables for the propensity score model (See Table A1). Variables were included in the propensity score model if they were plausible confounding candidates or were strongly related to the outcome. We excluded variables that could act as mediating variables of the effect of UFMs on meal costs, such as participation rates. We also excluded variables that were a subset of other variables, such that we used the Healthy Eating Index for lunch and breakfast separately, while excluding variables that measured whether the levels of a specific nutrient met nutrition guidelines. Finally, if any variables were correlated at 0.70 or higher, we excluded the variable with a lower correlation with the outcome. When possible, ordinal variables were treated continuously. We included 37 predictor variables in the propensity score model predicting universal free lunch (Table A1). We included breakfast-related variables in this propensity score model, but did create a separate set of weights for universal free breakfast or CEP participation. We used a cutoff point of 0.25 for the weighted standard mean difference between treatment and control covariates and a variance ratio of >2 to determine whether the propensity score model achieved balance [24].

We used the school-level analysis cost study weights, strata, and PSU adjusting for sampling and non-response separately for lunch and breakfast models. These were designed to provide nationally representative school-level cost data and be suitable for analysis of the relationship between school characteristics and meal costs [21]. These were reweighted using IPTW derived from the propensity score model.

We estimated the full and reported cost per lunch and per breakfast in separate survey-adjusted generalized linear regression models with a log-link function and gamma distribution. These models are frequently used to estimate healthcare costs, as they are both unbiased and precise estimators of the mean and accurate for regression parameters given the highly skewed distributions seen in cost data [25]. While the meal cost data were very skewed, they did not contain any zero-cost outcomes that would require two-part models or other approaches to deal with zero inflation. The primary predictor for these regressions was universal free lunch or universal free breakfast, respectively. In sensitivity analyses, we used school-level CEP participation as the primary predictor. We included an interaction term between the primary predictor and school size (schools with fewer than 500 students vs. larger schools) based on our hypothesis that cost savings due to economies of scale achieved by increased participation would be more likely to accrue for larger schools. Other covariates included the following: region, FRP meal eligibility category, availability of competitive foods during meals, school urbanicity, school type (elementary, middle, high), use of a food service management company, the number of categories of food service operations training received, and the HEI scores for the lunches and breakfasts served during the target survey week. In sensitivity analyses, we separately estimated models with an interaction between the primary predictor and FRP meal eligibility or urbanicity, respectively. In a separate sensitivity analysis, we restricted the analytic sample to schools with FRP meal eligibility ≥ 60%, and estimated the full and reported lunch cost using the same approach as the primary analysis. This analysis was designed to assess how sensitive our findings were to incorrect sample restriction due to the lack of data on school- or district-level ISP prior to weighting with the propensity score model.

We estimated marginal predictive means and conducted F tests for the contrast by UFM status across levels of school size and over levels of other interaction terms in sensitivity analyses. In line with best practices for evaluating and presenting multiplicative interaction models, we presented all constitutive terms, refrained from interpreting the magnitude or significance of model parameters as independently meaningful with regard to conditional hypotheses, and presented the mean and uncertainty for predictive margins [26]. Data management and weighting were performed in SAS 9.4 (Cary, NC, USA). All other analyses were conducted in Stata 15.0 (College Station, TX, USA; 2016), and accounted for the complex survey design.

## 3. Results

The SNMCS included 310 SFAs and 972 schools in the cost study sampling frame, from which 286 (90% weighted response rate) SFA cost estimates and 880 (91% weighted response rate) school cost estimates were generated from responding SFAs and schools. We dropped schools (*n* = 4) with incomplete data on variables in our propensity score model, leaving 876 schools. After schools not eligible for the CEP were excluded (*n* = 368), we had the final sample of 508 schools (with 209 SFAs), including 103 schools with universal free lunch, 87 that participated in the CEP, and 16 that operated the NSLP under Provision 2 or 3. Of the 119 schools that operated universal free school breakfast, 87 participated in the CEP and 32 operated universal free breakfast under Provision 2 or Provision 3.

**Figure A1:**
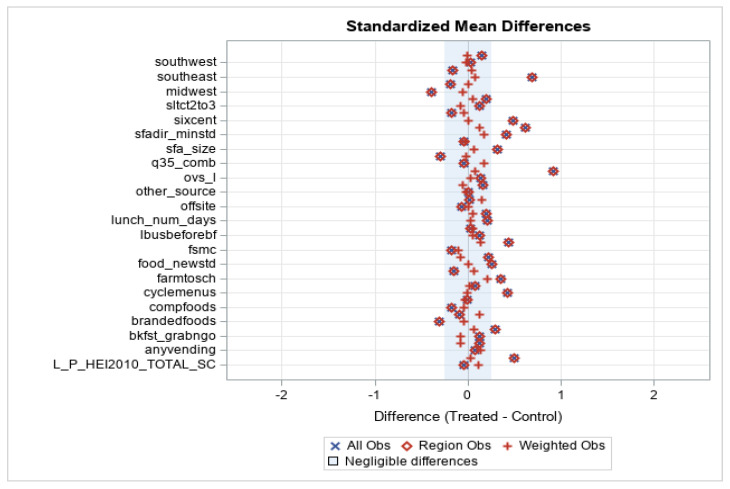
Standardized mean differences of predictors of universal free lunch participation before and after weighting from the propensity score model.

**Figure A2:**
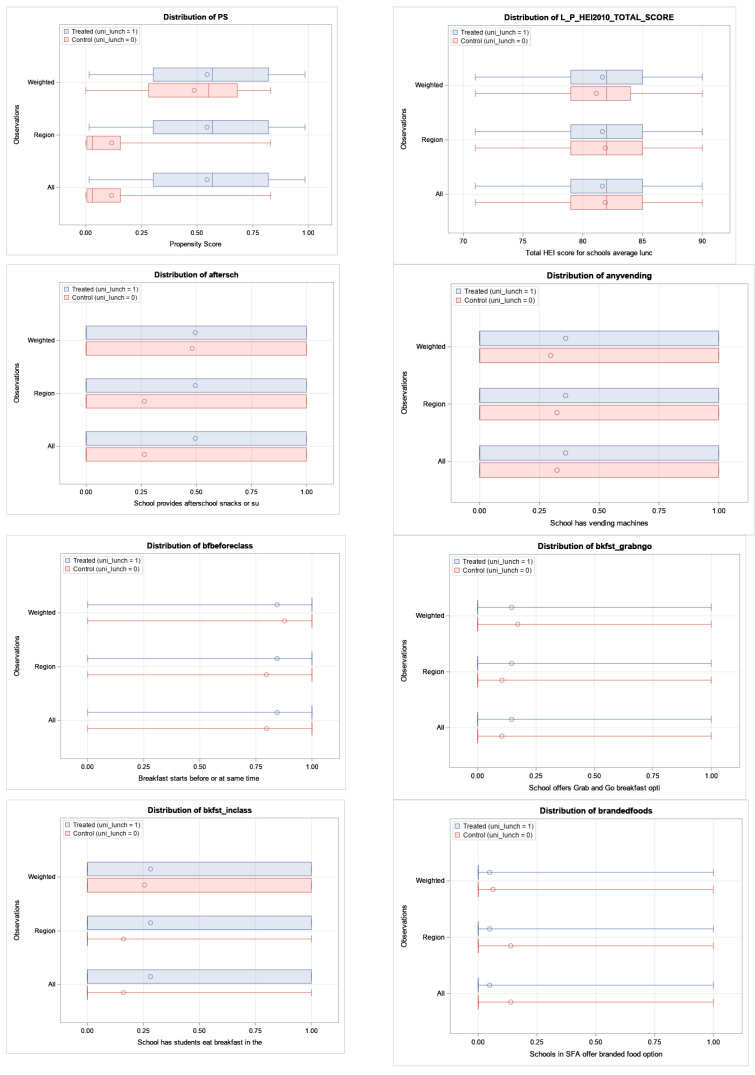

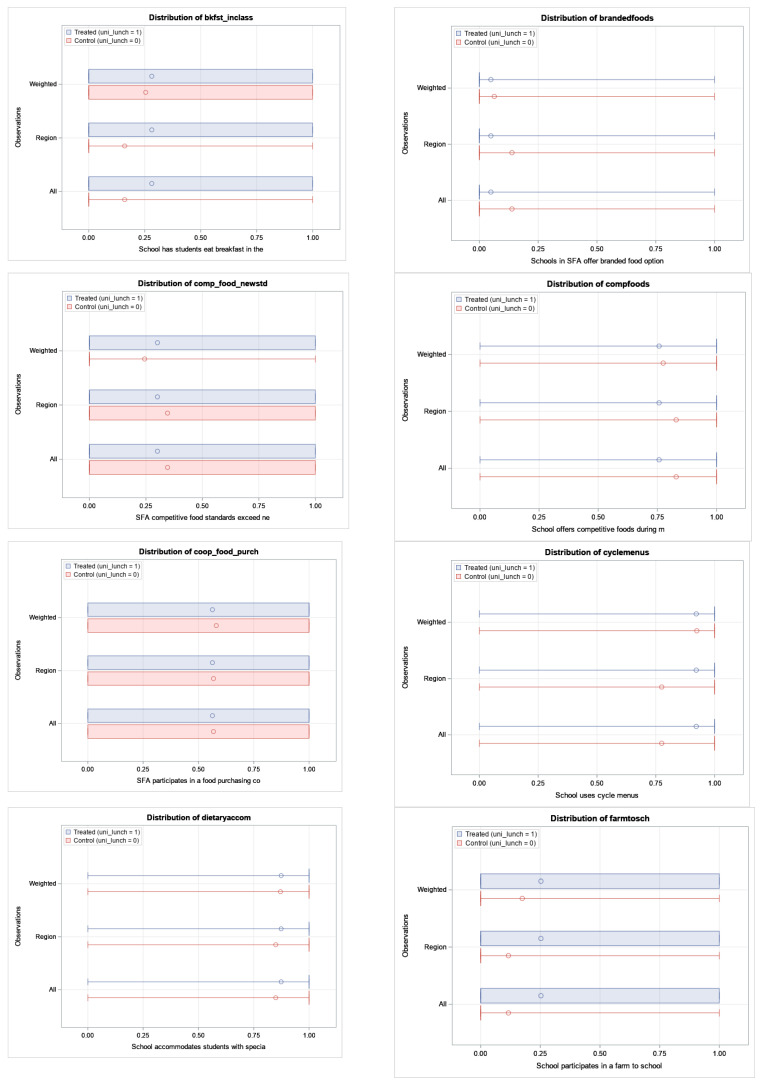

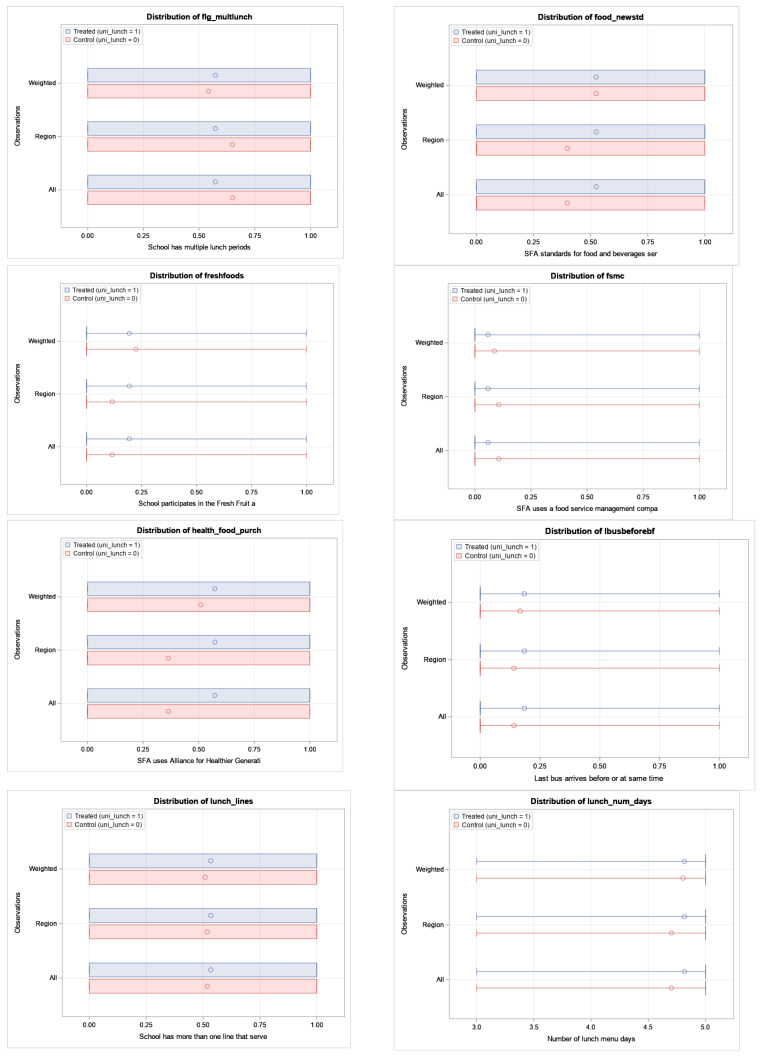

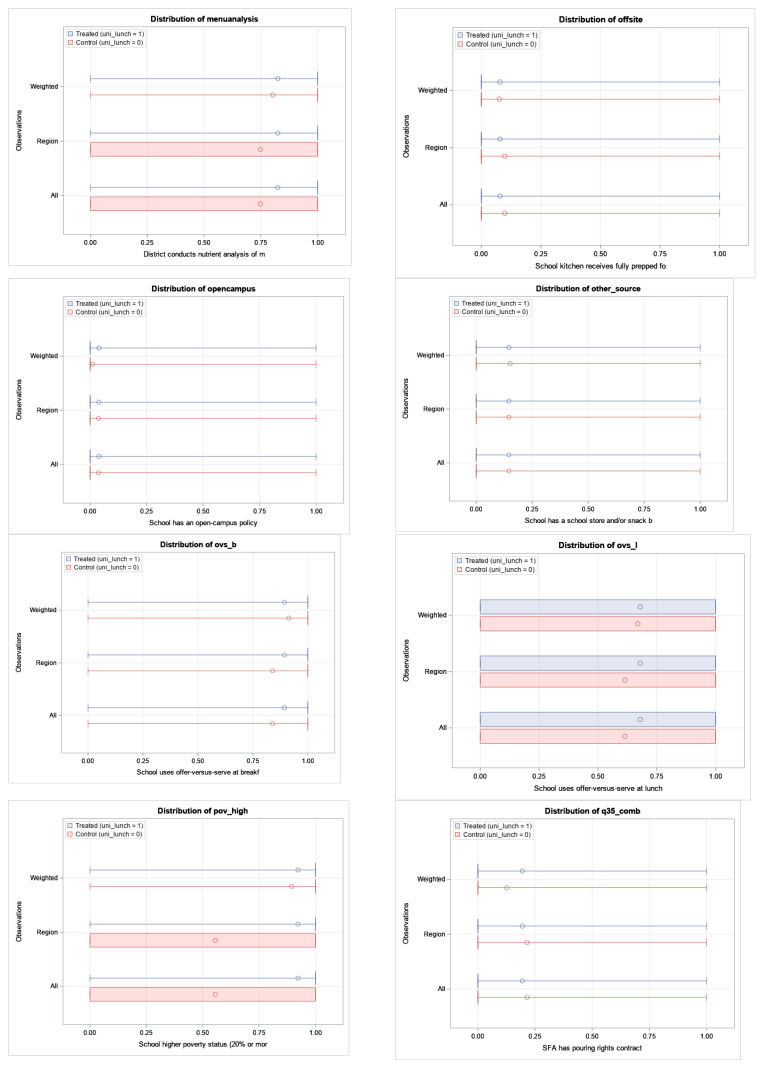

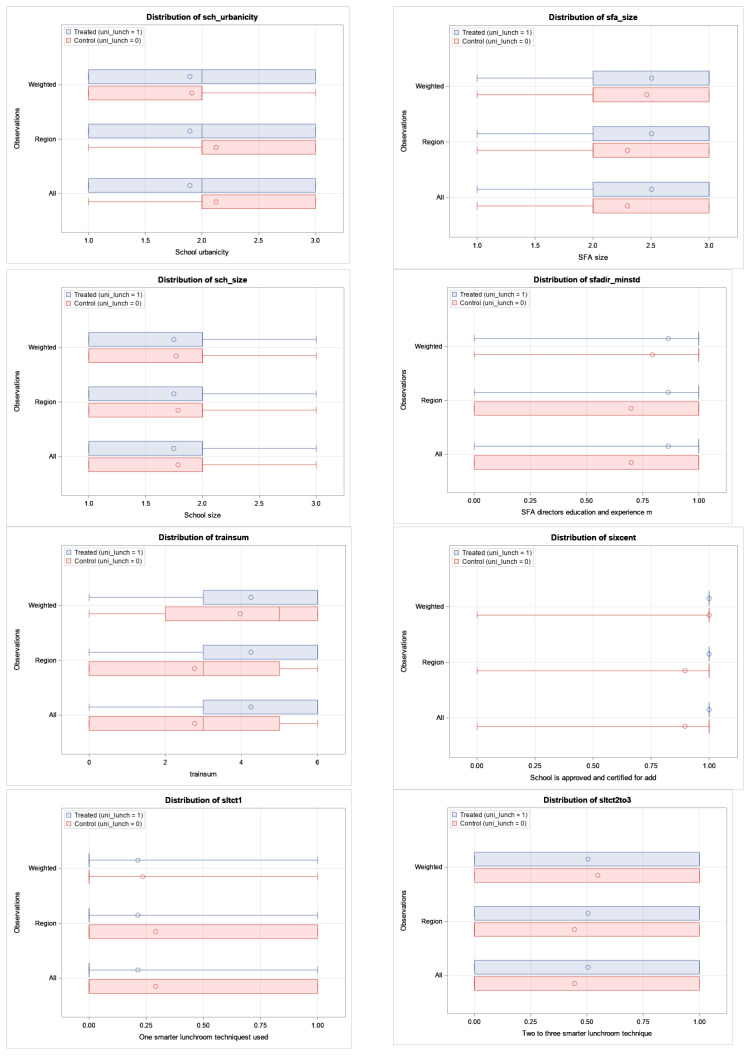

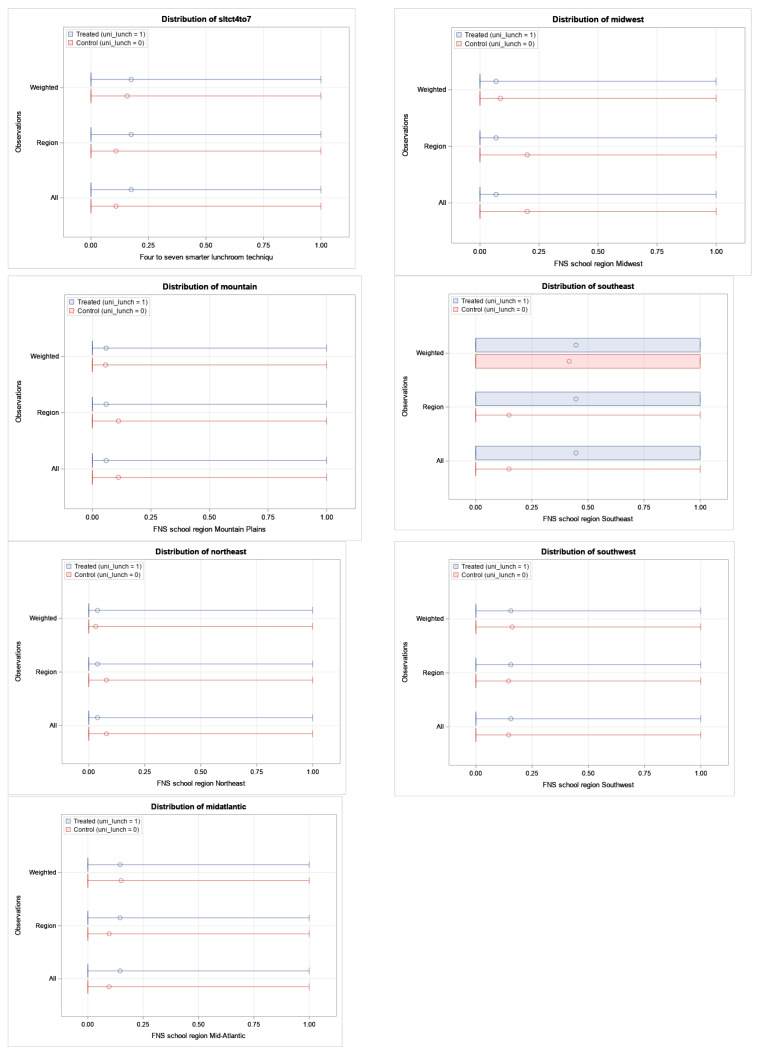
Propensity score and propensity score covariate weighted and unweighted distributions by universal free lunch status. Note: region and all observations are the same in this analysis.

The propensity score model was effective at balancing covariates (Table A1 and Figure A1). None of the standardized mean differences were above an absolute value of 0.25 [24]. The variance ratios for all but one predictor variable, including the propensity scores (open campus, variance ratio = 3.35), were below two. The mean difference and standardized mean difference for this variable were both small. We included the survey-adjusted and IPTW school characteristics for UFM participating schools and non-UFM participating schools (Table 1). We report the weighted and unweighted distributions for all variables used in the propensity score model in Figure A2.

**Figure 1:**
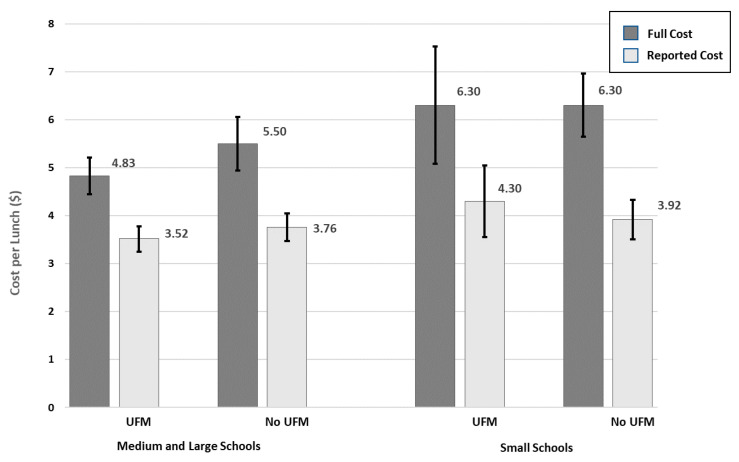
Generalized linear model predictive margin estimates of full and reported reimbursable school lunch cost by universal free lunch participation and school size.

Model parameters from the generalized linear models with a log-link function for the association between UFM (lunch and breakfast separately) and full meal costs are presented in Table 2. Model parameters using reported instead of full costs are presented in Table A3. The contrast of the predictive margins for full lunch cost by UFMs over school size showed no difference between UFM and non-UFM participating schools among small schools (−0.0002; 95% CI: −1.44, 1.44; F = 0.00 (1df), *p* = 0.998) and a marginally significantly lower full cost among medium/large schools participating in UFMs compared to medium/large schools not participating in UFMs (−0.673; 95% CI: −1.395, 0.0499; F = 3.37 (1df), *p* = 0.068). The predictive margin estimates of the mean full and reported lunch cost by UFM and school size are presented in Figure 1. Lunch costs were 13.9% higher among medium/large schools not participating in UFMs compared to medium/large schools participating in UFMs, and were only 6.8% higher when considering only reported costs. Reported costs accounted for 35.8% of the cost difference when using the full costs, so that unreported costs accounted for 64% of the total difference. Costs varied significantly across regions, and were lower for schools in districts using a food service management company and higher for schools with 80–100% FRP meal eligibility.

**Figure 2:**
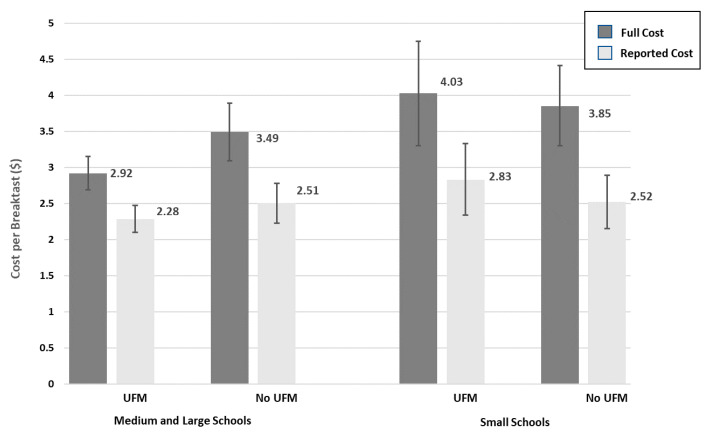
Generalized linear model predictive margin estimates of full and reported reimbursable school breakfast cost by universal free breakfast participation and school size.

Model parameters for the universal free breakfast model are presented in Table 2. Eight observations were excluded from this analysis due to missing data on the HEI score for breakfast. The contrast of the predictive margins for full breakfast cost by UFMs over school size showed no difference between UFM and non-UFM participating schools among small schools (0.171; 95% CI: −0.826, 1.169; F = 0.11 (1df), *p* = 0.735) and a substantially, statistically significant lower full cost among medium or large schools participating in UFMs compared to medium/large schools not participating in UFMs (−0.575; 95% CI: −1.077, −0.074; F = 5.11 (1df), *p* = 0.025). Predictive margins of the mean full and reported cost for breakfast by UFM participation and school size are presented in Figure 2. Consistent with findings for the lunch program, reported costs accounted for 40% of the cost difference associated with UFM participation among medium/large schools. For the breakfast model, training on more categories of foodservice practice was associated with significantly lower meal costs.

**Figure A3:**
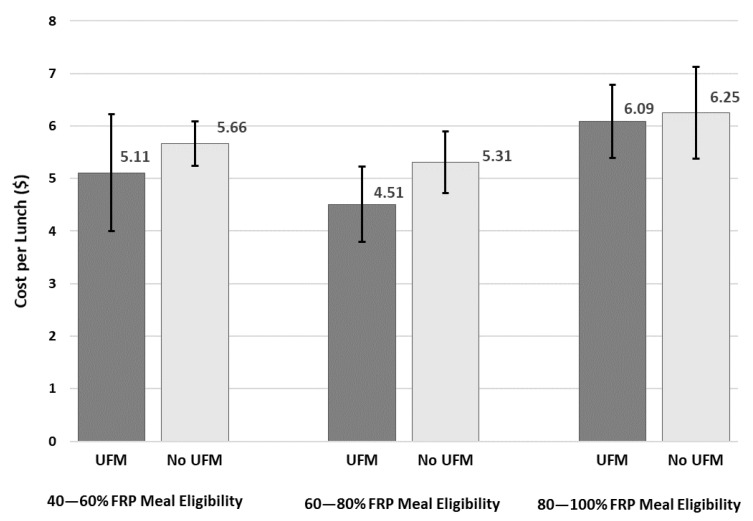
Generalized linear model predictive margin estimates of full reimbursable school lunch cost by universal free lunch participation and free and reduced price (FRP) meal eligibility.

**Figure A4:**
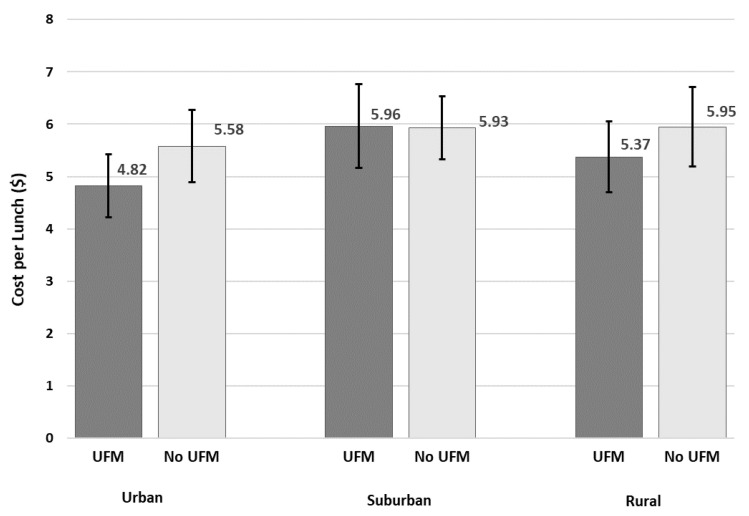
Generalized linear model predictive margin estimates of full reimbursable school lunch cost by universal free lunch participation and urbanicity.

In sensitivity analyses, we used CEP participation as the primary predictor. Results were largely similar, although the magnitude of the difference for breakfast was reduced and no longer significant in the contrast statement for medium/large schools participating in the CEP vs. not participating (−0.334; 95% CI: −0.838, 0.171; F = 1.70 (1df), *p* = 0.194) (Table A2). This difference highlights the relatively small sample of schools implementing the CEP or UFMs in this dataset. In an additional sensitivity analysis, we present the predictive margins for the full cost for lunch by UFM participation and FRP meal eligibility in Figure A3. Addressing whether our main findings were sensitive to misspecification of CEP eligibility due to a lack of data on ISP, we found larger cost savings among schools in the 40–60% and 60–80% FRP categories than among schools in the 80–100% FRP categories, although the differences were not statistically significant. This does not support the argument that our main analysis was sensitive to misspecification by including non-CEP eligible schools at the lower margin. We present the predictive margins for the full cost for lunch by UFM participation and urbanicity in Figure A4.

**Figure A5:**
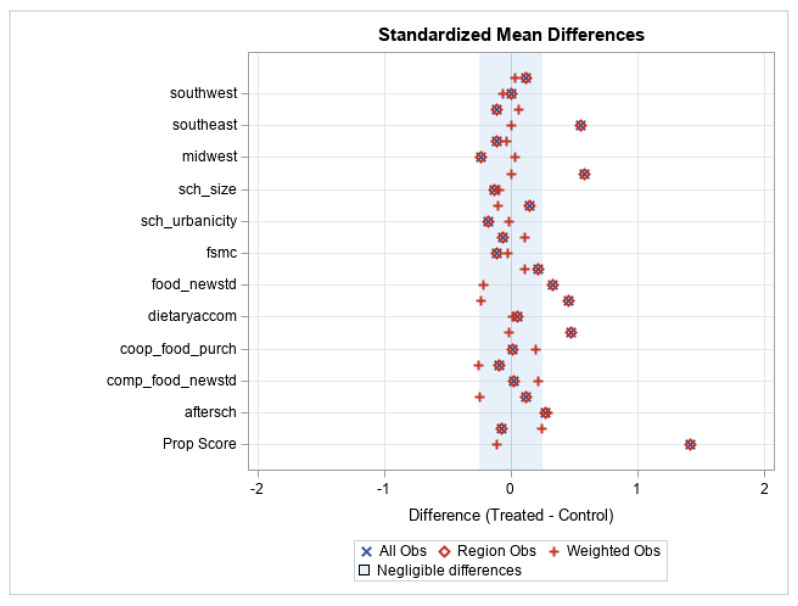
Standardized mean differences of predictors of universal free lunch participation before and after weighting from propensity score model in restricted sample of schools ≥ 60% free and reduced price meal eligibility (*n* = 284).

**Figure A6:**
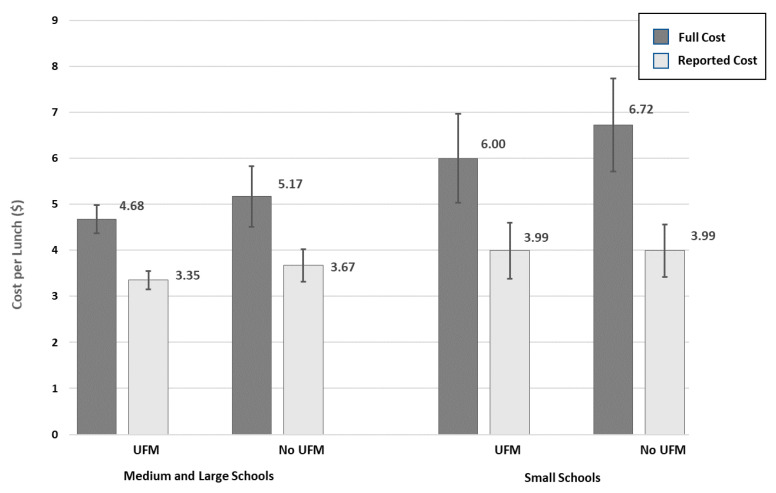
Generalized linear model predictive margin estimates of full and reported reimbursable school lunch cost by universal free lunch participation and school size in restricted sample of schools ≥ 60% free and reduced price meal eligibility (*n* = 284).

In the sensitivity analysis using the sample restricted to schools with FRP meal eligibility ≥ 60%, we included 284 schools (UFM *n* = 93, non-UFM *n* = 191). We re-estimated the PS model with a smaller set of covariates, finding an acceptable balance (Figure A5). We found that the relationship between UFMs and cost by school size in this sample was consistent with our primary analysis (Figure A6). The full cost per lunch among medium and large schools was 10.5% higher among non-UFM ($5.17) vs. UFM ($4.68), similar to 13.8% higher cost in the primary analysis. This difference was not significant in the restricted sample (F = 0.01 (1df), *p* = 0.916). While not significant in the smaller sample, the consistency of the findings with our main analysis does not support the argument of model misspecification by including non-CEP eligible schools at the lower margin.

## 4. Discussion

This study finds that participation in UFMs was associated with lower per-meal full costs in the SBP and meaningful, but marginally significant, lower costs in the NSLP among medium and large schools (over 500 students), suggesting that, at least in the first year of the CEP national implementation, the economy of scale was not yet available for CEP participating small schools. This occurs without any negative effect on the dietary quality of school meals, suggesting that UFMs can provide nutritious meals to more students without a financial disadvantage for UFM participating schools and school districts. We find no evidence that UFMs and CEP participation are negatively linked to the diet quality of school meals, despite lower per-meal costs.

The findings of lower per-meal costs among medium and large UFM schools are largely consistent with data from another study based on a large administrative 2010–2018 dataset of New York State schools and school districts. While this study uses a different design and cannot be compared directly, Rothbart et al. [19] used a difference-in-difference design to report the causal estimates of a decline in spending per meal following CEP implementation and an increase in federal reimbursements that more than offset the reduction in local food service revenue (with no collection of meal payments). Furthermore, the study also tested and rejected the hypothesis that expanding UFMs would crowd out education spending, finding no effect of CEP participation on instructional expenditures [19]. This analysis was outside the scope of our project, but future studies should aim to replicate the NYS study in other states and, ideally, national samples. Rothbart et al. found differential effects of CEP participation on meal service in rural areas, suggesting that the CEP increases school food program deficits in rural school districts by $30 per student [19]. We did not observe this finding in our national, cross-sectional sample, where meal costs did not appear to vary significantly by UFMs and urbanicity (Figure A4).

The SNMCS makes an important contribution to the study of school food finances in a number of ways. It is the largest nationally representative sample of schools and school food authorities reporting detailed revenue and expenses allocated to specific meal programs. The SNMCS also incorporates opportunity costs into the estimate of the cost per meal. These non-budgeted costs account for the time spent by district and school staff to help the program run. This accounting allows the field to move closer to the actual cost of running school meal programs, highlighting the extent to which school systems invest in meal programs beyond the reimbursement provided by the federal program. The median full cost per lunch in our analytic sample was $4.87, 52% higher than even the maximum reimbursement level for free lunches certified under HHFKA, which was $3.21 in SY 2014–2015 [27].

The improvements in full meal costs documented in this paper are similar to a broader trend in school food reform in the past two decades. We have previously reported that removing unhealthy competitive foods increases meal participation and is likely to improve or have no impact on the financial performance of school districts [28,29]. We found that reductions in school meal costs among medium and large schools (lunch (−$0.673; 95% CI: −1.395, 0.0499; *p* = 0.068) and breakfast (−$0.575; 95% CI: −1.077, −0.074; *p* = 0.025)) could be achieved with no change in the nutritional quality of meals and a potential improvement in student nutritional outcomes with the slightly higher meal participation rates from UFM participation. This suggests that a binary approach to balancing access to nutritious foods and maintaining financial performance may miss the potential of changing how meal programs are delivered to expand the set of efficient solutions. We found that almost two thirds of the difference between schools participating and not participating in UFMs came from unreported costs, suggesting that changing how the program is administered can lead to substantial efficiencies, even in the absence of economies of scale in the production of school meals.

The fast expansion of the CEP to 65% of eligible schools makes it essential that timely data on the effects of CEP participation are collected to guide decision makers in eligible schools and school districts. The COVID-19 pandemic has led to the implementation of UFMs across all schools nationwide, irrespective of student incomes [30]. This experience may lead to broader adoption of the CEP, which could be enhanced by changes in the rules and legislation supporting the program. Additional supports will be needed for smaller schools that might have more challenges in benefiting from economies of scale associated with UFMs and the CEP in particular.

### Strengths and Limitations

This study is based on cross-sectional data collected during the first year that the CEP became available nationwide. The sample of schools in 11 pilot states that implemented the CEP earlier (over 2011–2013) was too small to enable separate analyses. It is possible that the short-term effects observed in this study do not capture the full CEP effects that would be sustained over time. Longer-term studies are needed to replicate these results. Although this is the largest nationally representative school meal cost study to date, we did not have a large enough sample of UFM and non-UFM meals to utilize within-district variation in CEP or UFM implementation to exploit variation in district-level cost structures in our analyses. While we did control for region and urbanicity, we were not able to adjust for local- or state-level variations in costs. Although the response rate to the cost portion of the SNMCS was 90%, nonresponse bias remains a possibility that we were unable to assess with available data. Our study also had to rely on cross-sectional data, which precludes assessments of changes over time and limits causal inferences. We have attempted to address concerns related to the selection bias in choosing to participate in the CEP by inverse probability of treatment weighting (IPTW) derived from the propensity score model. While the use of IPTW achieved balance for a broad range of school- and SFA-level characteristics and propensity scores, the method is subject to bias from unobserved covariates and from potential violation of the positivity assumption, that all schools could have been assigned to UFM or no-UFM status. The lack of data on the ISP at the district and school level and our use of FRP meal eligibility categories to truncate the sample to ensure UFM eligibility is an imperfect solution. The SNMCS was designed as a nationally representative study for both district- and school-level analyses. Our subsample of the study restricted to schools likely to be eligible for the CEP or UFMs included more non-participating schools than national estimates (~80% vs. ~50%). This could be explained both by differences in the sampling frame given the smaller subset of the SNMCS overall sample used here and limitations in defining eligibility due to a lack of data on ISP. Our finding that the relationship between UFM participation and lower costs is in the same direction of all categories of FRP meal eligibility provides some confidence that the results would hold with a more severe truncation of the initial sample. We have assessed the relationship between UFMs and meal cost using both an interaction term with FRP and in a sample restricted to schools with higher FRP meal eligibility (≥60%), and found that relative reductions in cost were similar to our primary analysis. Given these findings, the lower level of participation in UFMs compared to the national level is likely a sampling issue, which does not threaten internal validity, but does limit generalizability. Additional research is needed into how ISP and FRP meal eligibility are related in national samples for the purpose of improving quasi-experimental studies of the impact of the CEP.

## 5. Conclusions

This paper provides evidence that UFM and CEP participation are associated with reduced per-student meal costs for medium and large schools, particularly for breakfast. These effects vary by school size, benefiting larger schools that can more effectively use the economies of scale from expanding access to meals to all students. These findings are limited by a lack of ISP data to determine eligibility and a small sample that may not generalize to all schools. The magnitude of potential cost savings from broader implementation and limitations of this study highlight the need for further research.

## Figures and Tables

**Table 1:** Survey-adjusted and inverse probability of treatment weighted characteristics of schools in the analytic sample from the School Nutrition and Meal Cost Study (*n* = 508).

Variable	Schools Participating in Universal Free Lunch Mean or % (95% CI) (*n* = 103)	Schools not Participating in Universal Free Lunch Mean or % (95% CI) (*n* = 405)
Region		
Mid-Atlantic	21.3 (0.0, 43.3)	11.4 (4.3, 18.4)
Midwest	10.4 (0.0, 23.8)	14.5 (8.9, 20.2)
Mountain	7.8 (0.0, 17.1)	11.9 (6.4, 17.4)
Northeast	2.3 (0.0, 6.7)	5.3 (2.0, 8.5)
Southeast	39.2 (11.9, 66.6)	21.2 (12.3, 30.1)
Southwest	9.6 (1.1, 18.1)	13.1 (7.4, 18.7)
West	9.4 (0.6, 18.3)	22.7 (14.5, 30.8)
School Size		
Small (<500 students)	31.3 (13.1, 49.5)	52.3 (44.4, 60.2)
Medium or Large (500 or more students)	68.7 (50.5, 86.9)	47.7 (39.8, 55.6)
Urbanicity		
Urban	33.2 (13.1, 53.4)	20.6 (12.8, 28.4)
Suburban	28.8 (5.7, 51.8)	46.3 (37.5, 55.1)
Rural	38.0 (9.8, 66.2)	33.1 (25.3, 40.9)
School Level		
Elementary School	61.1 (41.5, 80.7)	68.1 (63.9, 72.2)
Middle School	26.7 (9.7, 43.8)	18.2 (14.8, 21.6)
High School	12.2 (3.9, 20.4)	13.7 (11.5, 15.9)
FRP Meal Eligibility		
40–60%	9.1 (0.0, 19.1)	44.4 (36.7, 52.1)
60–80%	27.9 (0.0, 57.9)	32.2 (24.7, 39.7)
80–100%	63.0 (34.8, 91.2)	23.4 (16.0, 30.9)
Offers Competitive Foods During Meals	74.8 (58.2, 91.3)	79.4 (72.4, 86.3)
Uses Food Service Management Company	23.2 (0.0, 53.8)	9.1 (3.8, 14.4)
Number of Food Service Training Categories	2.5 (1.2, 3.8)	2.9 (2.4, 3.4)
HEI Score for Lunch	81.4 (80.2, 82.5)	81.9 (81.1, 82.7)

CI, confidence interval; SFA, School Food Authority; HEI, Healthy Eating Index; UFM, universal free meals; FRP, free and reduced price.

**Table 2:** Generalized linear model results for the association between universal free meals and full costs for the National School Lunch Program and School Breakfast Program.

Variable	Universal Free Lunch (*n* = 508)		Universal Free Breakfast (*n* = 496)	
	β (95% CI)	*p*-Value	β (95% CI)	*p*-Value
Constant	2.118 (1.153, 3.083)	0.000	1.668 (0.751, 2.584)	0.000
UFM	−0.130 (−0.268, 0.007)	0.063	−0.180 (−0.332, −0.028)	0.020
School Size				
Small (<500 students)	referent		referent	
Medium/Large (≥500 students)	−0.125 (−0.238, −0.011)	0.032	−0.121 (−0.273, 0.032)	0.121
Interaction: Small school × UFM	0.130 (−0.102, 0.363)	0.271	0.223 (−0.266, 0.473)	0.080
School Level				
Elementary	−0.031 (−0.117, 0.054)	0.473	−0.031 (−0.127, 0.064)	0.518
Middle School	referent		Referent	
High School	0.111 (−0.001, 0.222)	0.053	−0.025 (−0.136, 0.085)	0.652
Region				
Midwest	referent		referent	
Northeast	0.336 (0.135, 0.537)	0.001	0.141 (−0.094, 0.377)	0.237
Mid-Atlantic	0.185 (0.033, 0.338)	0.018	0.250 (0.078, 0.423)	0.005
Southeast	0.180 (0.006, 0.354)	0.043	0.334 (0.114, 0.554)	0.003
Southwest	0.080 (−0.086, 0.246)	0.344	0.178 (−0.058, 0.413)	0.138
West	−0.114 (−0.230, 0.002)	0.053	0.093 (−0.057, 0.244)	0.223
Mountain	0.035 (−0.096, 0.165)	0.603	0.133 (−0.516, 0.318)	0.157
Urbanicity				
Rural	Referent		referent	
Suburban	0.031 (−0.087, 0.150)	0.184	−0.088 (−0.230, 0.054)	0.225
Urban	−0.084 (−0.208, 0.040)	0.607	−0.058 (−0.220, 0.103)	0.478
FRP Meal Eligibility				
40 to 60%	Referent		Referent	
60 to 80%	−0.056 (−0.160, 0.0479)	0.289	−0.068 (−0.192, 0.055)	0.278
80 to 100%	0.133 (0.002, 0.263)	0.046	−0.057 (−0.193, 0.079)	0.408
Competitive food during meals	−0.0615 (−0.205, 0.082)	0.399	−0.095 (−0.192, 0.055)	0.270
SFA uses food service management company	−0.203 (−0.323, −0.083)	0.001	−0.001 (−0.167, 0.164)	0.988
Number of food service training categories	−0.010 (−0.031, 0.011)	0.335	−0.030 (−0.055, −0.005)	0.019
HEI Score for Lunch	−0.004 (−0.015, 0.007)	0.495	--	
HEI Score for Breakfast	--		−0.003 (−0.015, 0.009)	0.590

CI, confidence interval; UFM, universal free meals; FRP, free and reduced-price; SFA, School Food Authority; HEI, Healthy Eating Index.

## Data Availability

Requests for access to the public use version of these data should be submitted via electronic mail to FNSStudies@usda.gov.

## Appendix A

**Table A1:** Weighted standardized mean differences of predictors of universal free lunch participation from the propensity score model.

Variable	Mean Difference	Standardized Difference	Percent Reduction	Variance Ratio
**Model Specification**				
Propensity Score	0.05791	0.24770	86.48	1.4349
**Demographics**				
Region				
Mid-Atlantic	−0.00448	−0.01373	90.92	0.9741
Midwest	−0.01831	−0.05467	86.13	0.8025
Mountain	0.00216	0.00778	95.91	1.0349
Northeast	0.00779	0.03311	80.62	1.2392
Southeast	0.03026	0.06977	89.86	1.0158
Southwest	−0.00609	−0.01699	36.92	0.9680
West	--	--	--	--
School urbanicity	−0.01752	−0.02301	92.39	1.1910
School higher poverty status (20% or more)	0.02793	0.06983	92.39	0.7576
SFA size	0.04215	0.06443	79.79	1.2073
School size	−0.02081	−0.02873	44.67	0.9699
**Breakfast**				
Breakfast starts before or at start of first class	−0.03370	−0.08785	28.49	1.2265
School offers grab and go Breakfast	−0.02613	−0.07899	37.68	0.8735
Last bus arrives before or at the start time for breakfast	0.01723	0.04662	60.60	1.0789
Offer vs. serve at breakfast	−0.01978	−0.05816	63.16	1.1992
School has students eat breakfast in the classroom	0.02710	0.06579	77.62	1.0649
**Lunch**				
School has multiple lunch periods	0.03056	0.06269	60.08	0.9846
Average HEI total score from one week of lunches	0.50865	0.10554	0.00	0.9339
Number of lunch menu days	0.01236	0.02271	88.95	1.0187
Offer vs. serve at lunch	0.00950	0.01986	85.35	0.9838
**Competitive Foods**				
School has vending machines	0.06261	0.13173	0.00	1.1019
SFA competitive food standards exceed federal standards	0.05668	0.12094	0.00	1.1382
School has pouring rights contract	0.06829	0.16887	0.00	1.4202
School offers competitive foods during meals	−0.01733	−0.04283	76.05	1.0515
School has store or snack shop	−0.00588	−0.01662	0.00	0.9666
School has an open-campus policy	0.02758	0.14392	0.00	3.3503
**Nutrition Standards, Menu Planning, and Training**				
School uses cycle menus	−0.00244	−0.00695	98.34	1.0284
SFA meal standards exceed federal standards	0.00007	0.00015	99.94	0.9987
District conducts nutrient analysis of menus	0.02190	0.05354	71.60	0.9117
Number of food service training categories	0.28750	0.11987	80.57	1.0610
SFA director education and experience meets HHFKA standards	0.07031	0.17320	57.47	0.7165
**Operations**				
School provides afterschool snack or supper	0.01397	0.02954	93.95	1.0001
Schools in SFA offer branded foods	−0.01434	−0.04975	84.02	0.7828
SFA participates in food purchasing cooperative	−0.01749	−0.03518	0.00	1.0091
School accommodates students with special dietary needs	0.00320	0.00924	86.90	0.9777
School participates in a farm to school program	0.07845	0.20482	42.48	1.3115
School participates in fresh fruit and vegetable program	−0.02970	−0.08223	61.99	0.8994
SFA uses a food service management company	−0.02871	−0.10466	40.08	0.6900
SFA uses Alliance for Healthier Generation or similar purchasing tool	0.06298	0.12871	69.99	0.9780
School has more than one line for reimbursable meals or components	0.02460	0.04912	0.00	0.9945
School receives fully prepped foods from a central kitchen or production facility	0.00204	0.00719	90.32	1.0235
School is approved and certified for additional six-cent reimbursement	0.00000	0.00000	100.00	0.0000
One smarter lunchroom technique implemented	−0.02101	−0.04841	72.99	0.9343
Two to three smarter lunchroom techniques	−0.04318	−0.08637	28.52	1.0080
Four to seven smarter lunchroom techniques	0.01821	0.05229	72.45	1.0909

**Table A2:** Generalized linear model results for the association between community eligibility provision participation and full costs for the National School Lunch Program and School Breakfast Program.

Variable	Universal Free Lunch (*n* = 508)		Universal Free Breakfast (*n* = 496)	
	β (95% CI)	*p*-Value	β (95% CI)	*p*-Value
Constant	2.134 (1.168, 3.100)	0.000	1.730 (0.809, 2.652)	0.000
CEP Participation	−0.127 (−0.269, 0.015)	0.078	−0.106 (−0.263, 0.052)	0.188
School Size				
Small (<500 students)	referent		referent	
Medium/Large (≥500 students)	−0.134 (−0.243, −0.025)	0.017	−0.154 (−0.302, −0.006)	0.042
Interaction: Small school × UFM	0.112 (−0.157, 0.382)	0.82	0.187 (−0.090, 0.464)	0.184
School Level				
Elementary	−0.032 (−0.117, 0.052)	0.451	−0.035 (−0.129, 0.058)	0.459
Middle School	referent		referent	
High School	0.110 (−0.003, 0.223)	0.055	−0.031 (−0.141, 0.080)	0.586
Region				
Midwest	referent		referent	
Northeast	0.337 (0.137, 0.537)	0.001	0.154 (−0.082, 0.391)	0.200
Mid-Atlantic	0.188 (0.035, 0.340)	0.016	0.250 (0.076, 0.424)	0.005
Southeast	0.186 (0.008, 0.364)	0.041	0.332 (0.097, 0.567)	0.006
Southwest	0.081 (−0.086, 0.249)	0.341	0.191 (−0.042, 0.424)	0.108
West	−0.122 (−0.245, 0.002)	0.054	0.099 (−0.057, 0.255)	0.214
Mountain	0.035 (−0.093, 0.163)	0.592	0.132 (−0.054, 0.318)	0.164
Urbanicity				
Rural	referent		referent	
Suburban	0.034 (−0.085, 0.153)	0.577	−0.084 (−0.224, 0.056)	0.239
Urban	−0.085 (−0.213, 0.042)	0.189	−0.057 (−0.224, 0.110)	0.502
FRP Meal Eligibility				
40 to 60%	referent		referent	
60 to 80%	−0.060 (−0.163, 0.042)	0.249	−0.082 (−0.203, 0.040)	0.185
80 to 100%	0.132 (0.010, 0.254)	0.035	−0.089 (−0.228, 0.050)	0.207
Competitive food during meals	−0.061 (−0.163, 0.042)	0.249	−0.079 (−0.248, 0.090)	0.356
SFA uses food service management company	−0.203 (−0.323, −0.083)	0.001	−0.020 (−0.183, 0.143)	0.809
Number of food service training categories	−0.010 (−0.031, 0.011)	0.337	−0.029 (−0.054, −0.003)	0.027
HEI score for lunch	−0.004 (−0.015, 0.007)	0.480	--	
HEI score for breakfast	--		−0.004 (−0.016, 0.008)	0.492

CI, confidence interval; CEP, community eligibility provision; FRP, free and reduced price; SFA, School Food Authority; HEI, Healthy Eating Index.

**Table A3:** Generalized linear model results for the association between universal free meals and reported costs for the National School Lunch Program and School Breakfast Program.

Variable	Universal Free Lunch (*n* = 508)		Universal Free Breakfast (*n* = 496)	
	β (95% CI)	*p*-Value	β (95% CI)	*p*-Value
Constant	1.30 (0.448, 2.15)	0.003	1.201 (0.441, 1.960)	0.002
UFM	−0.067 (−0.186, 0.052)	0.267	−0.095 (−0.237, 0.047)	0.19
School Size				
Small (<500 students)	referent		referent	
Medium/Large (≥500 students)	−0.048 (−0.153, 0.056)	0.364	−0.049 (−0.202, 0.105)	0.532
Interaction: Small school*UFM	0.159 (−0.053, 0.370)	0.140	0.212 (−0.272, 0.452)	0.080
School Level				
Elementary	−0.056 (−0.145, 0.033)	0.217	−0.050 (−0.134, 0.034)	0.241
Middle School	referent		Referent	
High School	0.100 (−0.004, 0.203)	0.059	0.004 (−0.100, 0.107)	0.946
Region				
Midwest	referent		referent	
Northeast	0.128 (0.001, 0.254)	0.048	0.046 (−0.164, 0.256)	0.666
Mid-Atlantic	0.052 (−0.063, 0.166)	0.375	0.182 (0.060, 0.303)	0.004
Southeast	0.176 (0.011, 0.341)	0.037	0.354 (0.139, 0.569)	0.001
Southwest	0.040 (−0.109, 0.190)	0.593	0.216 (−0.014, 0.447)	0.066
West	−0.026 (−0.149, 0.096)	0.673	0.227 (0.106, 0.347)	<0.001
Mountain	0.017 (−0.106, 0.140)	0.784	0.131 (−0.031, 0.292)	0.113
Urbanicity				
Rural	Referent		referent	
Suburban	0.060 (−0.057, 0.177)	0.316	−0.088 (−0.202, 0.075)	0.370
Urban	−0.023 (−0.138, 0.092)	0.695	−0.045 (−0.197, 0.106)	0.555
FRP Meal Eligibility				
40 to 60%	Referent		Referent	
60 to 80%	−0.054 (−0.146, 0.037)	0.242	−0.047 (−0.153, 0.060)	0.388
80 to 100%	0.022 (−0.101, 0.146)	0.724	−0.099 (−0.225, 0.026)	0.121
Competitive food during meals	−0.083 (−0.200, 0.035)	0.169	−0.096 (−0.243, 0.050)	0.195
SFA uses food service management company	−0.149 (−0.255, −0.042)	0.006	0.102 (−0.080, 0.283)	0.271
Number of food service training categories	−0.012 (−0.032, 0.011)	0.234	−0.024 (−0.050, 0.002)	0.075
HEI score for lunch	0.001 (−0.008, 0.011)	0.759	--	
HEI score for breakfast	--		−0.005 (−0.013, 0.007)	0.527

CI, confidence interval; UFM, universal free meals; FRP, free and reduced price; SFA, School Food Authority; HEI, Healthy Eating Index.
